# Cells of origin of lung cancers: lessons from mouse studies

**DOI:** 10.1101/gad.338228.120

**Published:** 2020-08-01

**Authors:** Giustina Ferone, Myung Chang Lee, Julien Sage, Anton Berns

**Affiliations:** 1 Division of Molecular Genetics, The Netherlands Cancer Institute, 1066 CX Amsterdam, The Netherlands;; 2 Department of Pediatrics, Stanford University School of Medicine, Stanford, California 94305, USA;; 3 Department of Genetics, Stanford University School of Medicine, Stanford, California 94305, USA

**Keywords:** LuADC, LuSCC, NSCLC, cell of origin, lung cancer, mouse models

## Abstract

As one of the most common forms of cancer, lung cancers present as a collection of different histological subtypes. These subtypes are characterized by distinct sets of driver mutations and phenotypic appearance, and they often show varying degrees of heterogenicity, aggressiveness, and response/resistance to therapy. Intriguingly, lung cancers are also capable of showing features of multiple subtypes or converting from one subtype to another. The intertumoral and intratumoral heterogeneity of lung cancers as well as incidences of subtype transdifferentiation raise the question of to what extent the tumor characteristics are dictated by the cell of origin rather than the acquired driver lesions. We provide here an overview of the studies in experimental mouse models that try to address this question. These studies convincingly show that both the cell of origin and the genetic driver lesions play a critical role in shaping the phenotypes of lung tumors. However, they also illustrate that there is far from a direct one-to-one relationship between the cell of origin and the cancer subtype, as most epithelial cells can be reprogrammed toward diverse lung cancer fates when exposed to the appropriate set of driver mutations.

## Abstract (precis)

In this review, Ferone et al. summarize the work performed in experimental mouse models of lung cancer that specifically sheds light on the cell of origin of lung cancers.

Lung cancer is the world's deadliest cancer. Every year, more people die of lung cancer than of colon, breast, and prostate cancers combined (Ferlay et al. 2015; Adjei 2019). While some ascribe the deadliness of lung cancers to the fact that the disease is often detected at a more advanced stage, the main difficulty in improving patient survival lies in that lung cancer is a very challenging cancer to treat. Surgery can be performed in early-stage disease. However, once the cancer has disseminated within the lung and metastasized to other tissues, systemic treatments are often the only option. Lung cancers come in multiple flavors, and it is crucial to tailor the treatment according to the subtype. We can distinguish lung cancers into roughly two major subgroups: nonsmall cell lung cancer (NSCLC), which accounts for ∼85% of cases and is further subdivided into lung adenocarcinoma (LUAD) and lung squamous cell carcinoma (LSCC), as well as large cell carcinoma (LCC), and small cell lung cancer (SCLC), which accounts for the remaining 15% of lung cancer cases (Siegel et al. 2017).

Of all the major subtypes of lung cancer, LUAD patients benefit from the largest selection of treatment options. LUAD patients receive combinations of chemotherapy, targeted therapy (e.g., inhibitors of EGFR and ALK), and immunotherapies according to their genotypic and phenotypic stratification, resulting in a substantial survival benefit for a subset of patients (Ramalingam and Belani 2008; Han et al. 2015; Xia and Herbst 2016; Hida et al. 2017; Peters et al. 2017). Treatment options are much more limited for LSCC (∼30% of lung cancers) and SCLC patients for whom almost no effective treatment option substantially extending survival has become available over the last 25 yr. Most LSCC and SCLC patients succumb to their disease within a few months or a few years. Recently, immunotherapy has shown long-term benefit in a small fraction of LSCC and SCLC patients (Brahmer et al. 2015; Kogure et al. 2018; Armstrong and Liu 2019). Other innovative therapies are being explored but so far without significant breakthroughs.

To develop more effective therapies and improve patient survival, we must gain a better understanding of the biology of these diseases, with the hope of finding new vulnerabilities and identifying widely applicable screening markers that permit earlier detection of these cancers. This is a challenging task, as illustrated by the slow advance of such developments in LSCC and SCLC. LSCC can present with very diverse sets of driver lesions including a substantial contribution of tumor suppressor gene losses, making it difficult to identify suitable targets for therapeutic intervention (The Cancer Genome Atlas Research Network 2012). Similarly, SCLC, with predominant loss of both *RB1* and *TP53* tumor suppressor genes, does not exhibit strong driver pathway dependencies for which drugs are available (George et al. 2015). Although the frequent amplification and overexpression of MYC family members might make them attractive therapeutic targets, we currently lack effective drugs against them in the clinic. Similarly, targeting the frequently amplified and overexpressed BCL2 protein or the activated PI3K pathway has yielded disappointing results besides being associated with significant toxicity (Tarhini et al. 2010; Baggstrom et al. 2011; Langer et al. 2014). Furthermore, the significant heterogeneity of lung tumors often resulting from long-term carcinogen exposure and chromosomal instability has resulted in tumor populations in which escape mutations are often abundantly present.

This raises the question of whether other treatment paradigms that do not exclusively depend on the acquired oncogenic lesions but take advantage of specific characteristics of the cancer cell of origin could serve as a way forward. The use of rituximab in the treatment of non-Hodgkin's lymphoma (NHL) and other hematopoietic malignancies (Mohammed et al. 2019) provides an example for such an approach, where lineage-specific cell surface markers serve as therapeutic target to eradicate tumor cells that belong to a specific hematopoietic lineage. Furthermore, heterogeneous populations arising from a different cell of origin even within the same tumor subtype may also determine clinically relevant features such as local dissemination, metastatic potential, and response to therapy and therefore serve as a predictive marker. Consequently, defining the cell of origin can help undercover the mechanisms of tumor initiation and progression and identify unique cell type-specific targets for therapy (Visvader 2011; Blanpain 2013). Assessing the cells of origin of human lung tumors has proven difficult as these tumors usually have a long history of accumulating driver and passenger mutations that, together with environmental factors, can impact tumor development. The presence of markers characteristic for lung epithelial cell subtypes can be used to infer a cell of origin for that tumor, whether it is LUAD (Tabbo et al. 2018) or SCLC (Rudin et al. 2019). However, ongoing single-cell sequencing and 3D organoid approaches are likely to help achieve a much better understanding of the early stages of lung cancer development in humans in the future. Back and forth studies between mouse models and human analyses probably offer the best perspectives for studying prevention, early detection, and more effective treatment paradigms.

In this review, we summarize the work performed in model systems of lung cancer that specifically sheds light on the cell of origin of lung cancers. We chose to review here mostly studies performed in mice, as this approach permits a more thorough analysis of the specific location and features of early lesions. We refrain from including studies that do not address cell of origin aspects of tumor development and response/resistance to therapy.

## Epithelial lineages in the lung

**Figure 1.**
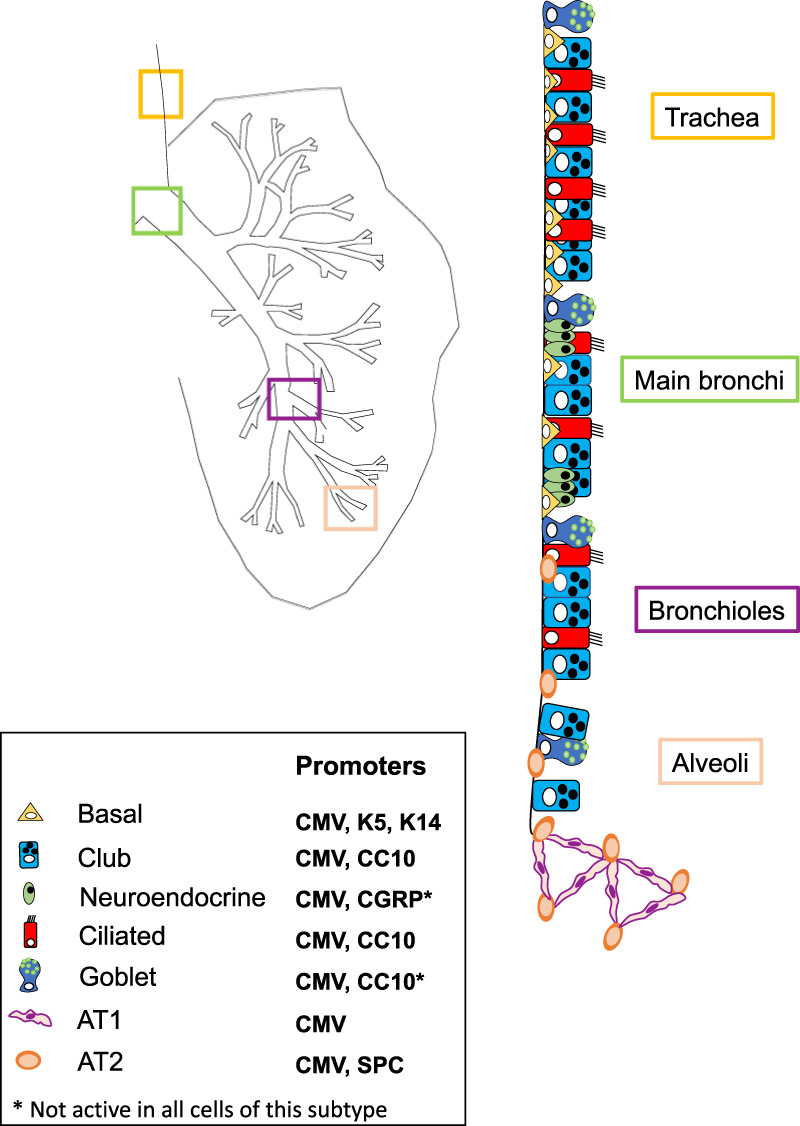
Schematic representation showing how mouse lung composition varies from the trachea to the alveolar space. Basal, club, neuroendocrine and AT2 cells are the major differentiated subtypes and have been either engineered or targeted to express tumor driver mutations.

The lung is a complex organ composed of many different cell types. In contrast to some other tissues that show very high rates of turnover, such as the hematopoietic system and the intestinal tract, the turnover of lung tissue is relatively slow, with a turnover time of 7 yr in humans. However, upon injury, the tissue has the capacity to quickly repair the damage through the mobilization of resident cells with tissue stem cell properties (Rawlins and Hogan 2006; Kim 2017; Leach and Morrisey 2018; Lee and Rawlins 2018). These specialized cells, such as basal cells and subsets of alveolar type II (AT2) cells, are capable of giving rise to the diverse lineages that line the different anatomical compartments of the respiratory system. The major differentiated cell subtypes in the lung are represented by their localization and role in maintaining the lung structure: Alveolar type I (AT1) and II (AT2) cells are responsible for forming and maintaining the alveolar structures, with the AT1 cells being responsible for gas exchange; the club and ciliated cells cover the trachea and bronchi along with the basal epithelial cells lining the basement membrane; and a numerous range of more specialized cells are distributed both dispersed and at specific locations (e.g., at bronchi bifurcation sites or in the transition from the bronchioles to the alveoli). Among these rarer cell types are the innervated neuroendocrine cells important for gauging intrapulmonary small molecule levels and controlling the biochemical milieu by the regulated secretion of a range of bioactive peptides. Neuroendocrine cells are present both as clusters (as neuroepithelial bodies present mostly at bifurcation sites) and dispersed single cells throughout the trachea and bronchi (see Fig. 1; Garg et al. 2019).

## GEMMs as a tool for uncovering cellular mechanisms of lung cancer development

Most of the knowledge about lung development and how it is controlled by specific transcriptional programs comes from studies using genetically engineered mouse models (GEMMs). Similarly, GEMMs have been heavily used to increase our understanding of what drives the development of the various lung cancer subtypes (see Tables 1–3). While studying human lung cancers offers unique challenges such as the difficulty in dissecting the specific factors responsible for cancer initiation, GEMMs can alleviate some of these challenges by offering a system in which individual gene expressions may be tweaked. Advanced mouse models of lung cancer allow for both spatial and temporal control of oncogene activation and tumor suppressor inhibition (Kwon and Berns 2013; Sánchez-Rivera and Jacks 2015). Because mice used to model lung cancer live under controlled genetic and environmental conditions, the development of lung tumors is highly reproducible, allowing for studies of cancer initiation and progression that are still impossible to model in patients. Mouse models also provide a powerful system to investigate epigenetic heterogeneity within tumors or during cancer progression (Tammela and Sage 2019). Perhaps most importantly, however, mouse models can provide a phenotypic readout when genetic modulations of interest are introduced in specific cell lineages. As we argue further below, it is the combination of both the specific genetic lesions and the cell of origin that determines tumor characteristics.

**Table 1.**
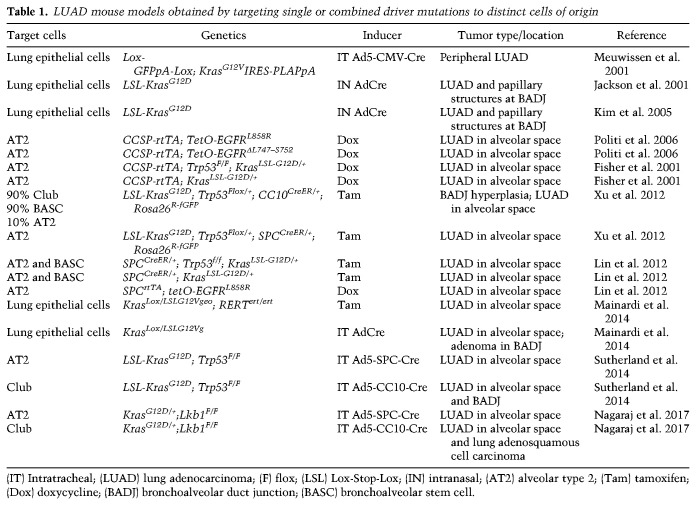
LUAD mouse models obtained by targeting single or combined driver mutations to distinct cells of origin

**Table 2.**
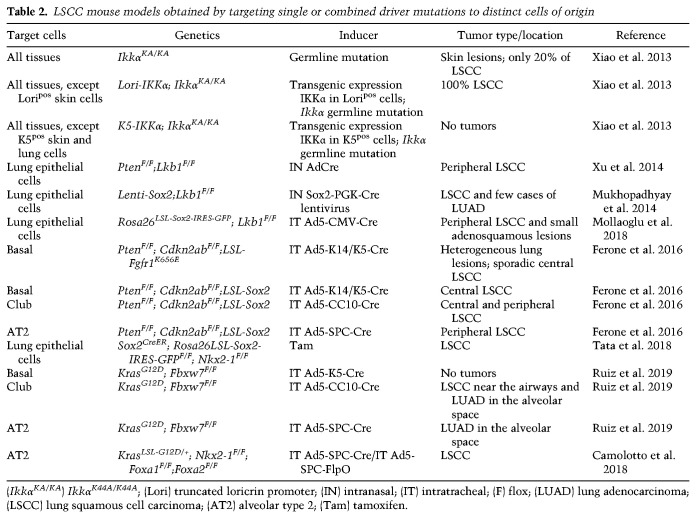
LSCC mouse models obtained by targeting single or combined driver mutations to distinct cells of origin

**Table 3.**
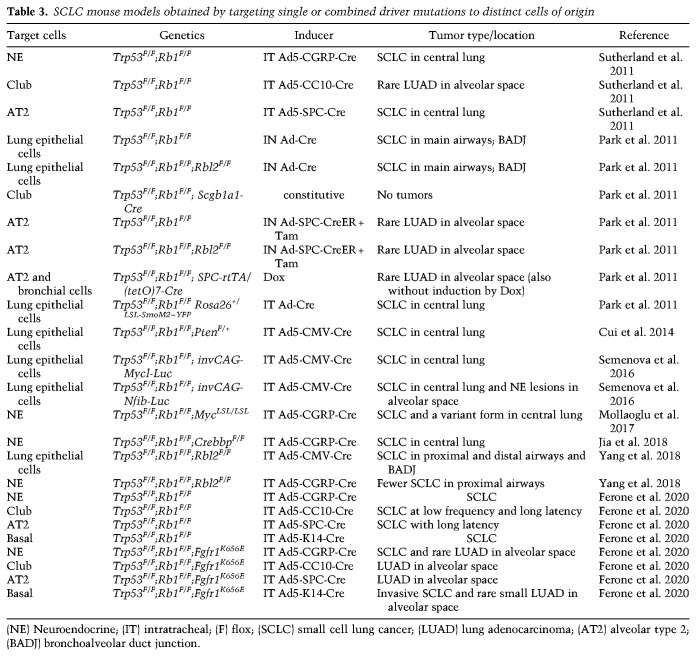
SCLC mouse models obtained by targeting single or combined driver mutations to distinct cells of origin

So far, the approach to identify a cell of origin for cancers has been limited to the use of cell type-specific Cre drivers either by using engineered knock-in strategies or by infecting lung cells with adenoviruses driving Cre expression by specific promoters. Each of these approaches has their own advantages and limitations, and we deal with these in the context of their specific application.

Here we further explore to what extent the combination of the cell of origin and the set of distinct mutations determines specific tumor features such as its location, morphology, microenvironment, plasticity, latency, heterogeneity, and aggressiveness.

## Cell of origin of lung adenocarcinoma (LUAD)

**Figure 2.**
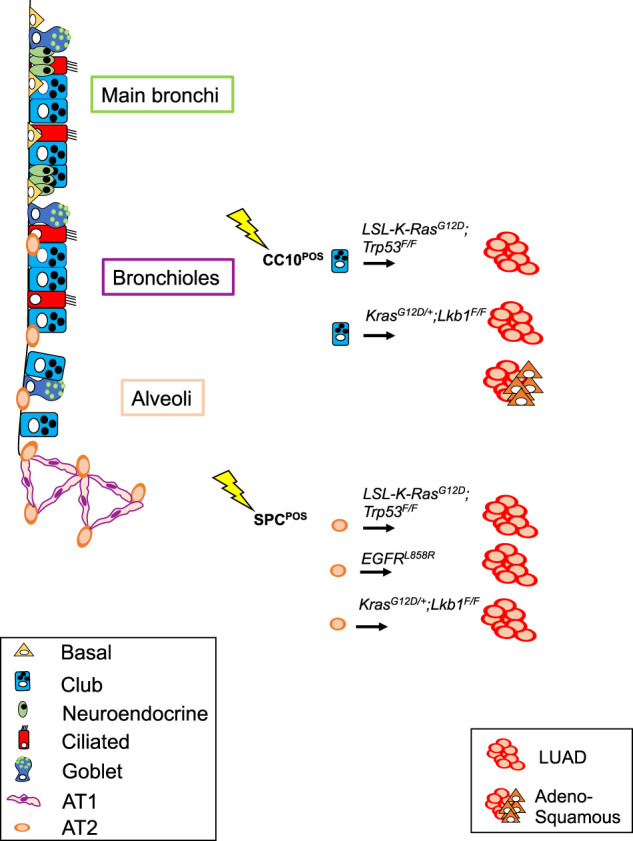
Schematic representation of genetic lesions that have resulted in LUAD in mouse models. Targeted cells of origin throughout the lung are also shown.

Lung adenocarcinoma (LUAD) (Fig. 2) is the most common type of lung cancer. A large proportion of the cases are caused by tobacco smoking, which is responsible for causing base-substitutions in cancer-related genes such as *TP53* and *KRAS*. Still, nonsmokers represent ∼25% of all lung cancer cases, the vast majority of which is LUAD. In these cases, LUAD often presents with point mutations in *EGFR* and specific gene fusions (e.g., *ALK*, *ROS1*, and *RET*) (Sun et al. 2007; The Cancer Genome Atlas Research Network 2014). Other commonly inactivated tumor suppressor genes include *KEAP1*, *STK11*, and *NF1* (Sun et al. 2007; The Cancer Genome Atlas Research Network 2014).

Since accumulating evidence suggested activating mutations in *KRAS* as a key initial event in LUAD tumorigenesis, conditional mutant *Kras*^*G12D*^ has been expressed in various mouse lung compartments using multiple approaches with the aim to identify the cells of origin of LUAD. A transgenic mouse model permitting spatio-temporal induction of sporadic activation of mutant *Kras* was generated by Meuwissen et al. (2001). These transgenic mice (*β-Actin*-*Lox-GFP-Stop-Lox-KRAS^G12V^-IRES-PLAP*) expressed ubiquitous GFP while a polyadenylation signal prevented mutant *KRAS* expression. The expression of KRAS^G12V^ could subsequently be induced along with placental alkaline phosphatase (PLAP) upon intratracheal delivery of adenoviral Cre. Mice infected with adenovirus carrying Cre under transcriptional control of the cytomegalovirus promoter (Ad5-CMV-Cre) showed progressive LUAD with a short latency (5–8 wk). The development of LUAD at the lung periphery (intraparenchymal lesions) and the absence of bronchial adenocarcinoma in spite of the efficient targeting of bronchial epithelial cells suggested that AT2 cells serve as the most prominent cell of origin of *KRAS*^*G12V*^-induced LUAD. Several mutant *Kras* knock-in models were generated by the Jacks laboratory: one in which spontaneous sporadic activation of mutant *Kras* did occur (Johnson et al. 2001) and the widely used *Lox-Stop-Lox-Kras^G12D^* knock-in model (Jackson et al. 2001), where the mutant *Kras* was induced upon intranasal instillation of recombinant adenoviral Cre. Mice in this latter model developed LUAD within 14 wk. Due to the presence of papillary structures located at the bronchiole/alveoli border at the end of a stretch of club cells, which express both the club cell marker CC10 and the AT2 cell marker SPC, the authors suggested a model in which *Kras*^*G12D*^ promoted the transdifferentiation of club cells into CC10–SPC-double-positive cells, which serve as the cell of origin of lung adenoma and adenocarcinoma (Jackson et al. 2001). Using this same model, Kim et al. (2005) demonstrated that these double-positive cells were also present in normal lung. These cells, which showed self-renewal capacity and were multipotent in clonogenicity assays, were named bronchioalveolar stem cells (BASCs). However, more recent studies have demonstrated that CC10^+^ club cells and SPC^+^ AT2 cells, rather than the double-positive BASCs themselves, serve as the cell of origin for LUAD upon *Kras*^*G12D*^ activation (Xu et al. 2012). Upon tamoxifen administration, *LSL-Kras^G12D^; Trp53^F/+^; CC10^CreER^; Rosa26^R-fGFP^* mice (where F stands for “floxed,” meaning that part of the gene is flanked by *loxP* sites) developed bronchioalveolar duct junction (BADJ) hyperplasia and LUAD in the alveolar space. In spite of Cre activation in nearly 90% of club cells (Rawlins et al. 2009), no tumors were found in the bronchi and upper airways, suggesting that club cells have a different susceptibility to LUAD transformation. They also pointed to the presence of CC10^+^ AT2 cells capable of initiating LUAD. To investigate whether only CC10^+^ AT2 cells were capable of transformation, Xu et al. (2012) administered tamoxifen to *LSL-Kras^G12D^; Trp53^F/+^; Sftpc^CreER^*; *Rosa26^R-fGFP^* mice and found that SPC^+^CC10^−^ alveolar cells were also efficiently transformed into LUAD. BADJ cells remained largely unaffected, even if they appeared labeled. Overall, their results suggested that both SPC^+^CC10^+^ and SPC^+^CC10^−^ cells in the alveoli can serve as the cells of origin of LUAD.

As BASCs and some of the other lung epithelial populations are activated to proliferate and repopulate the lung in response to injury, one may hypothesize that relative cellular plasticity and activation in response to injury could enhance development of lung cancer. Indeed, Mainardi et al. (2014) demonstrated that the adenoviral infection itself contributed to the permissiveness of lung cells to become transformed into LUAD. These investigators used a model in which mutant *Kras*, along with the *Bgeo* marker, was induced by 4-OH-tamoxifen treatment, which activates CreER expression from the *RERT* locus encoding the large subunit of RNA polymerase II. In this model, mice developed malignant LUAD in the alveolar space 24 wk after Cre activation. Targeted and stained cells at other lung sites did not expand further beyond a small cluster of cells. When Cre was intratracheally delivered via an adenoviral vector, mice developed papillary hyperplasia at the BADJ region that progressed to adenomas expressing both CC10 and SPC markers but not to malignant tumors. Only adenomas in the alveolar space, positive for SPC and not for CC10, progressed to malignant tumors. Therefore, both the target cell and the way in which mutations are activated can affect the permissiveness for tumor formation. Importantly, a number of lung cell populations aside from BASCs has been implicated in undergoing transdifferentiation in response to concomitant inflammation or local damage, such as the differentiation of club cells to AT2 cells (Zheng et al. 2013).

Another study that interrogated whether LUAD can arise from multiple cells of origin was based on the selective targeting of either CC10^+^ or SPC^+^ cells by using highly specific lineage-restricted recombinant Cre adenoviruses (Sutherland et al. 2014). *Kras^LSL-G12D/+^* mice developed LUAD following infections with either of the viruses. However, the tumors in these mice showed different localization and exhibited a distinct phenotype. Following Ad5-SPC-Cre infection, the mice developed tumors exclusively in the alveolar space but not in the BADJ region. Tumors were positive for SPC but not for CC10, in line with previous observations by Xu et al. (2012). Ad5-CC10-Cre infection of *Kras^LSL-G12D/+^* mice resulted in papillary hyperplasia at the BADJ, which involved not only CC10^+^SPC^+^ BASCs but also CC10^+^ club cells. Lineage tracing experiments with Ad5-CC10-Cre-injected *LacZ* mice did not reveal the previously reported CC10^+^ AT2 cells in the alveolar space (Rawlins et al. 2009); therefore, either club cells or BASCs, both present in the BADJ region, could have served as the potential cells of origin for LUAD. By using *Kras^LSL-G12D/+^;R26R-Confetti* mice, it was shown that CC10^+^ hyperplasic cells gradually lose the expression of CC10 and gain expression of SPC, resulting in SPC^+^ adenomas (Sutherland et al. 2014). This suggested that CC10^+^SPC^−^ populations served as the cell of origin of this subset of adenomas exhibiting a more papillary phenotype. The fact that Ad5-SPC-Cre infection did not promote LUAD in the BADJ region suggested that BASCs were not the cell of origin of LUAD. In a model with both *Trp53* deletion and mutant *Kras*^*G12D*^, LUAD originated from both Ad5-CC10-Cre or Ad5-SPC-Cre injected mice; in this setting, the tumor development was accelerated, and mice developed metastasis (Sutherland et al. 2014), indicating that different cells can function as the cell of origin of LUAD with carcinomas originating from club cells exhibiting more pronounced papillary features.

In conclusion, this group of studies has pointed to AT2 cells as the predominant cell of origin of LUAD (Mainardi et al. 2014). This holds true for mutant KRAS-induced as well as mutant EGFR-induced tumors with or without concomitant loss of *TP53* (Fisher et al. 2001; Politi et al. 2006) Furthermore, most studies have excluded BASCs as a cell of origin of LUAD, based on the absence of detectable LUAD development at the BADJ upon targeting (Lin et al. 2012).

A recent mouse model combined *Kras*^*G12D*^ and loss of the tumor suppressor *Lkb1* (also known as *Stk11*) in club and AT2 cells by using either Ad5-CC10-Cre or Ad5-SPC-Cre viruses (Nagaraj et al. 2017). Co-occurring *KRAS* mutations with *LKB1* deletions are found in ∼30% of human LUAD patients and are responsible for an aggressive form of a metastasis-prone NSCLC subtype. Modeled in mice, the cell of origin appears to influence the survival and histopathology spectrum of the *Kras^G12D^;Lkb1^Δ/Δ^* driven tumors. Ad5-SPC-Cre-injected mice exhibited a longer latency to tumor development than Ad5-CC10-Cre-injected mice and only developed typical LUAD; meanwhile, Ad5-CC10-Cre injected mice developed acinar and mucin types of LUAD and, more importantly, lung adenosquamous carcinoma. This suggests that the role of LKB1 is restricted to airway cells, and therefore its loss in alveolar cells does not significantly affect LUAD originating from AT2 cells. However, other studies (Han et al. 2014) have provided evidence that LUAD initiated from *Kras^G12D^;Lkb1^Δ/Δ^* mutant AT2 cells tend to transdifferentiate to an adenosquamous phenotype, suggesting that features seemingly imposed by the cell of origin can be modulated by other, so far not defined, factors.

The most relevant mouse models of LUAD with relevant information about the nature of the genetic lesions, the cell type specificity of the mutation inducer, and the tumor location are summarized in Table 1 (see also Fig. 2). The findings demonstrate that multiple cell types in the lung can give rise to LUAD. Furthermore, the effects of driver lesions are also dependent on the cell of origin, even to the extent that LUAD initiated from the same set of driver lesions result in tumors with different characteristics depending on whether AT2 or club cells were targeted. The heterogeneity resulting from the cell of origin is likely further amplified by intratumoral heterogeneity, in which tumors display hierarchical features as driven by Wnt signaling-mediated paracrine interactions (Tammela et al. 2017).

Therefore, it will be important to build models to assess more systematically how LUAD development hijacks distinct signaling pathways in their cells of origin that play a critical role during normal lung development and tissue renewal. MAPK signaling in AT2 cells is exemplary in this respect (Desai et al. 2014), making these cells specifically vulnerable for mutant EGFR- and KRAS-mediated transformation. The same holds for Wnt signaling (Nabhan et al. 2018), which may play a more important role in LUAD (Tammela et al. 2017) than previously suspected. The knowledge acquired by these studies might enable us to stratify patients with apparently the same histotype as well as provide inroads to new therapeutic strategies. For instance, can the basal cells serve as the cell of origin of LUAD if reprogrammed with the appropriate set of drivers, and how would response to therapy of these tumors differ from that of LUAD initiated from club cells? Given that a substantial fraction of cancer patients present with mixed histology or show evidence of transdifferentiation from one cancer subtype to another (e.g., LUAD to SCLC) following treatment, plasticity is a hallmark of malignant lung cancers. It is therefore worthwhile to investigate whether these cancers nevertheless retain distinct cell of origin features that can serve as target for intervention.

## Cell of origin of lung squamous cell carcinoma (LSCC)

**Figure 3.**
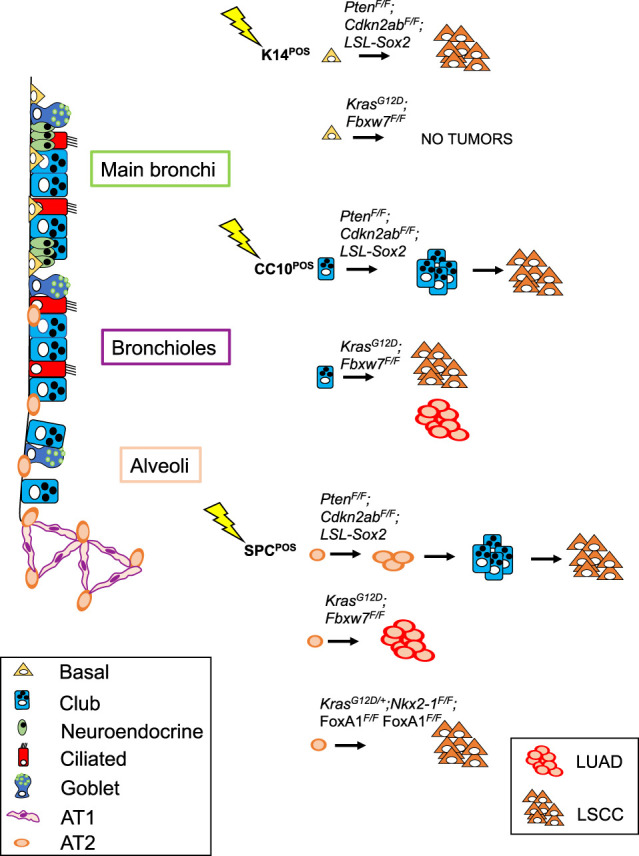
Schematic representation of genetic lesions that have resulted in LSCC in mouse models. Targeted cells of origin throughout the lung are also shown.

It has long been hypothesized that LSCC (Fig. 3) arises from tracheobronchial basal cells, which is in line with the notion that well-differentiated LSCC expresses more or less homogenously p63 and keratins K14 and K5, the markers of tracheobronchial basal cells that are not expressed in the peripheral lung (Cole et al. 2010; Travis et al. 2011; Vaughan et al. 2015). Therefore, LSCC is expected to develop predominantly in the upper airways. However, it appears that peripheral LSCC occurs almost as frequently as central LSCC (Funai et al. 2003; Sakurai et al. 2004; Yousem 2009; Hayashi et al. 2013). Accordingly, GEMMs in which either basal or alveolar cells are targeted have shown to mimic human central and peripheral LSCC, although with variable efficiency and with an important role played by the driver mutations.

In the LSCC models first described, the cell of origin was not unequivocally defined: This is the case for the IKKα knock-in mouse model (Xiao et al. 2013), as well as for the *Lkb1^F/F^;Pten^F/F^* and *Lenti-Sox2;Lkb1^F/F^* mouse models (Mukhopadhyay et al. 2014; Xu et al. 2014). IKKα acts in the NF-κB pathway but also serves as a switch controlling differentiation of epithelial cells (Descargues et al. 2008), whereas LKB1 acts in the AMPK pathway regulating cell growth and energy metabolism (Li et al. 2015). SOX2 is a transcription factor critical for conferring stem/progenitor cell features (Laughney et al. 2020). The IKKα model was generated by introducing a germline mutation in which lysine residue at amino acid 44, an ATP-binding site, was substituted to alanine to produce kinase-dead *Ikkα* knock-in (*Ikkα^K44A/K44A^*) (Xiao et al. 2013), as *IKKα* was found to be disrupted in a small percentage of human LSCC (1.7% according to TCGA). In mice, disruption of IKKα activity led to SCC development in lung and skin epithelia. Re-expression of the wild-type IKKα in K5-expressing cells prevented SCC development in both tissues. Since the genetic alteration was not somatically inducible in a spatio-temporal fashion, it remains unclear to what extent the disruption of IKKα activity in all cells throughout development influences disease development and even the cell of origin. However, the observation that SCC development is prevented by the K5-specific expression of IKKα clearly supports a basal–epithelial cell as the likely cell of origin in this specific model (Xiao et al. 2013).

In 2014, two conditional LSCC mouse models were described based on *Lkb1* loss (Mukhopadhyay et al. 2014; Xu et al. 2014), which is found in ∼2% of human LSCC (The Cancer Genome Atlas Research Network 2012). One model combined *Lkb1^F/F^* and *Pten^F/F^* conditional tumor suppressor alleles that were inactivated by intranasal (IN) delivery of adenovirus with Cre-recombinase under a ubiquitous promoter; in this model, the mice developed LSCC in the peripheral lung with a latency of 40–50 wk (Xu et al. 2014). Delivery of adenovirus with Cre under surfactant protein C promoter (SPC-Cre) or club cell secretory protein promoter (CC10-Cre) both failed to induce tumors when used with *Lkb1^F/F^* and *Pten^F/F^* conditional alleles. No more specific information on the cell of origin was reported with this set of tumor suppressor disruptions, although the data indicated that these genetic lesions were unable to initiate LSCC from AT2 and club cells. In the other model, a lentiviral approach was used to drive the expression of *Sox2* and Cre-recombinase in a *Lkb1^F/F^* conditional mouse strain (*Lenti-Sox2;Lkb1^F/F^*). Mice developed LSCC and, in a few cases, LUAD with a latency of 6–10 mo and 40% penetrance (Mukhopadhyay et al. 2014). It is possible that *Sox2* expression and *Lkb1* loss (Han et al. 2014) enable the reprogramming of distinct lung cell lineages to a squamous-like identity, but since the lentivirus used did not act in a specific cell type, this study did not provide further insight into the cell of origin of LSCC.

A recent study presented a more detailed analysis of *Lkb1* loss-based models and described squamous tumors and small adenosquamous lesions with predominantly peripheral localization at early time points, suggesting that these tumors originated from the distal lung epithelium (Mollaoglu et al. 2018). Differently from previous work (Mukhopadhyay et al. 2014), overexpression of SOX2 was not mediated by lentiviral delivery but from the *Rosa26* locus (Mollaoglu et al. 2018). In this study, Mollaoglu et al. (2018) suggested that SOX2 overexpression following Ad5-CMV-Cre injection makes the AT2 cells permissive to squamous differentiation due to both SOX2-mediated NKX2-1 suppression and recruitment of tumor-associated neutrophils (TANs). In this context, the development of LSCC is driven by the cell of origin, the genetic drivers, and interactions between the cancer cells and immune cells. Interestingly, LUAD induced by *Kras*^*G12D*^ can progress to LSCC upon subsequent deletion of *Lkb1*, which appears to cause loss of PRC2 through reduced expression of EED, a critical component of the PRC2 complex thereby permitting lineage switching (Zhang et al. 2017).

To develop a model of LSCC carrying the genetic lesions most frequently found in human LSCC, Ferone et al. (2016) generated mouse models based on the biallelic deletion of both *Pten* and *Cdkn2ab*, two genes frequently inactivated in human LSCC. However, this combination alone was insufficient to promote LSCC: Mice developed heterogeneous lesions 10–15 mo following Ad5-K14-Cre intratracheal delivery. Therefore, *Pten* and *Cdkn2ab* biallelic inactivation was combined with the overexpression of either *Fgfr1* or *Sox2* genes, which are frequently amplified in human LSCC (The Cancer Genome Atlas Research Network 2012). When combined with conditional overexpression of a constitutive active form of *Fgfr1* (*Fgfr1*^*K656E*^ allele) in basal cells, sporadic LSCC within heterogeneous lesions were found after a latency of 2–5 mo. The most successful inducer of squamous cell fate appeared to be *Sox2* overexpression in combination with *Pten* and *Cdkn2ab* deletions (*Sox2PC* mice). This combination was sufficient to transform either K14^+^ or K5^+^ basal cells into LSCC with 100% penetrance and a latency of ∼7 mo. In addition, findings in *Sox2PC* mice showed that club and AT2 cells were also efficiently reprogrammed toward a squamous fate, giving rise to LSCC with the same penetrance and latency as observed for basal cells (Ferone et al. 2016). Interestingly, targeting this set of driver lesions to AT2 cells showed that during their transition to LSCC, the AT2 cells first started to express the club cell marker CC10 while losing the AT2 marker SPC and subsequently acquired basal cell markers p63 and K5 with the concomitant loss of the transiently expressed CC10. When the lesions were targeted to club cells, they lost CC10 expression while acquiring the specific markers for LSCC. These results indicate that a combination of lesions often found in human LSCC can effectively induce LSCC from all the major lung cell types. However, the data also show that even a strong driver such as *Sox2* overexpression (Lu et al. 2010), alone or in combination with a single additional driver lesion, cannot transform a nonsquamous cell. For instance, the combined loss of the lung identity transcription factor *Nkx2-1* and *Sox2* overexpression promoted LSCC when switching was directed to airway epithelial cells but not when targeted to AT2 cells (Tata et al. 2018).

Thus, although lung cells show substantial plasticity and can be reprogrammed with a combination of three different genetic lesions, the cellular and epigenetic context determines the distinct combinations of driver lesions that may effectively cause transformation of that particular cell. Apparently, the combination of *Sox2* overexpression with concomitant loss of *Pten* and *Cdkn2ab* is able to release this epigenetic restriction in multiple cell lineages in the lung.

Recently, specific sets of mutations were shown to drive a mixture of LSCC and LUAD in a model based on *Kras*^*G12D*^ activation in combination with *Fbxw7* deletion (*KF* mice), a gene that codes for a ubiquitin ligase that targets several well-known oncoproteins (Ruiz et al. 2019). In this case, predominance of one or the other histotype was dictated by the cell of origin that was targeted. Targeting *Fbxw7^Δ/Δ^* and *Kras*^*G12D*^ specifically to K5-expressing cells failed to give rise to LSCC. In contrast, *KF* mice infected with Ad5-CC10-Cre virus developed tumor lesions with histological characteristics of LSCC that were mostly located in and adjacent to the airways. Ad5-CC10-Cre infection also resulted in 20% of LUAD tumors, which were found exclusively in the alveolar space. Targeting AT2 cells with Ad5-SPC-Cre in *KF* mice resulted exclusively in adenomas and adenocarcinomas distributed over the alveolar area. Whereas LUAD tumors in the *KF* mouse model originated from SPC^+^ AT2 cells, LSCC tumors originated from CC10^+^ luminal cells of the airways. This finding further underscored the importance of the cell of origin in determining lung cancer subtype development even in the presence of the same genetic lesions.

On the other hand, other studies of LSCC highlighted the role of specific genetic drivers in shaping the subtype determination of lung cancers. *Kras*^*G12D*^ activation with concomitant *Nkx2-1* deletion (*Kras^LSL-G12D/+^; Nkx2-1^F/F^*) together with either *Foxa1* or *Foxa2* disruption was reported to promote squamous differentiation of tumor lesions (Camolotto et al. 2018). These mice developed LUAD juxtaposed to LSCC lesions (adeno-squamous lesions), and further examination revealed that while the LUAD lesions were genetically proficient for either *Foxa1* or *Foxa2*, the squamous compartment was actually negative for the expression of both. This result suggested that the expression of either FOXA1 or FOXA2 was required in the initiation phase but that the subsequent loss of both was necessary for the cells to undergo squamous differentiation in this genetic background. In contrast, when both *Foxa1* and *Foxa2* were genetically deleted from the beginning, mice developed LUAD expressing markers of the squamo–columnar junction of the gastrointestinal tract. The investigators suggested that this difference was attributable to a context-specific regulation of lung cancer identity by NKX2-1, FOXA1, and FOXA2. By using sequential in vivo recombination, they showed that *FOXA1/2* loss in established KRAS-driven neoplasia originating from SPC^+^ alveolar cells was capable of promoting keratinizing squamous cell carcinomas, illustrating the capacity of these transcription factors to cause transdifferentiation. However, since these mutations were not induced in other lung cell lineages, this leaves open the possibility that this set of mutation can also promote LSCC in other lung compartments.

Key mouse models of LSCC are summarized in Table 2 (see also Fig. 3), with relevant information about the nature of the genetic lesions, the cell type specificity of the mutation inducer, and the tumor location. From the studies listed above, a number of important conclusions can be drawn:

(1) The various lung lineages show extensive plasticity. LSCC can be induced from basal epithelial cells, AT2 cells, and club cells. A limited set of driver lesions frequently found in human LSCC is sufficient in mice to give rise to LSCC from these different cell types, suggesting that those cell types might serve as the cell of origin of LSCC in humans also.

(2) Transformation of the different lung cells as described for *Sox2PC* mice (Ferone et al. 2016) results in LSCCs with indistinguishable expression profiles, suggesting profound reprogramming. Whether any unique epigenetic markers of the cell of origin are retained has not been studied and would be worth further exploring.

(3) The order in which the mutations accumulate matters. The observations made by Snyder and coworkers (Camolotto et al. 2018) are very intriguing in this respect. They illustrate that genetic lesions (such as loss of *FOXA1/2*) can inhibit LSCC if occurring early on by commanding a shift in cellular identity. However, they do promote LSCC when occurring during later phases of tumor development.

## Cell of origin of small cell lung cancer (SCLC)

**Figure 4.**
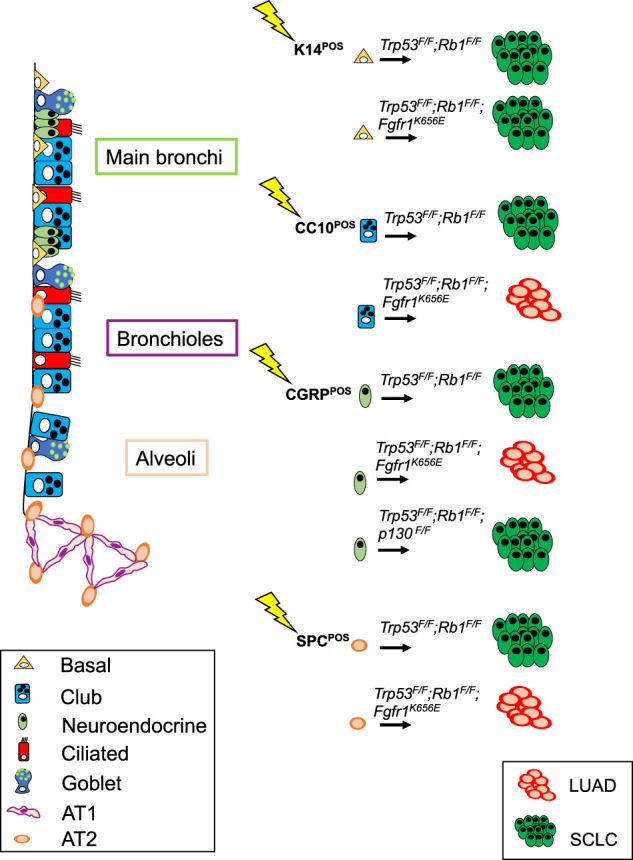
Schematic representation of genetic lesions that have resulted in SCLC in mouse models. Targeted cells of origin throughout the lung are also shown.

Accounting for ∼15% of all lung cancer cases, SCLC (Fig. 4) is a common and particularly lethal form of neuroendocrine (NE) lung cancer (Sabari et al. 2017). Several subtypes of SCLC have been recently classified on the basis of transcription factor expression patterns (Rudin et al. 2019). The major subtypes are characterized by the predominant expression of either the ASCL1, NEUROD1, POU2F3, and YAP1 transcription factors. To what extent the cell of origin is correlated with these subtypes is still an intriguing but unresolved question. The available data suggest that both the driver lesions set as well as the cell of origin will command the tumor subtype (Ireland et al. 2020). All SCLC subtypes are currently treated in the first-line with platinum-based chemotherapies, radiotherapy, and immunotherapy (Calles et al. 2019); however, resistance to treatment ensues rapidly, illustrating the urgent need to develop alternative treatment options. SCLC shows near-ubiquitous loss of function of the *RB1* and *TP53* tumor suppressor genes (George et al. 2015), but a better characterization of the genetic drivers of this cancer and the contribution of its potential cells of origin to tumor characteristics are needed for developing more effective therapies.

SCLC was initially thought to arise exclusively from the NE lung epithelial cells, a rare population of cells in the lung. However, experiments conducted in *Trp53^F/F^;Rb1^F/F^* mice by Sutherland et al. (2011) showed that cell type-restricted adenoviral vectors carrying Cre under the promoter of the *SPC* gene (Ad5-SPC-Cre), enabling deletion of *Rb1* and *Trp53* tumor suppressor genes specifically in AT2 cells, was also able to induce SCLC, although much less efficiently as compared with Ad5-CGRP-Cre that directed Cre expression to CGRP^+^ NE cells. Ad5-CC10-Cre (targeting club secretory cells) was very inefficient to induce SCLC, indicating that club cells are relatively resistant to transformation to neuroendocrine cancers in this genetic context. All tumors developed by targeting CGRP^+^ NE cells were located in the central region of the lung, as is the case for most SCLCs in human, were highly invasive, and almost exclusively belonged to the *Ascl1^+^* subtype. Although these results confirmed that NE cells are likely the predominant cell of origin for SCLC, they also suggested that AT2 cells are capable of giving rise to SCLC. In a way, this is reminiscent of the transdifferentiation of LUAD into SCLC, seen with a low but significant incidence in EGFR-mutant LUAD patients treated with EGFR inhibitors (Niederst et al. 2015). Apparently, the down-regulation of RAS signaling creates a condition that facilitates transdifferentiation to SCLC, provided that RB is inactivated. A similar phenomenon is observed in prostate cancer, emphasizing the almost absolute requirement for RB loss in SCLC as well as the notion that strong MAPK signaling is not well tolerated in SCLC (Calbo et al. 2011). Other studies were in line with the notion that NE cells serve as the primary cell of origin of SCLC (Park et al. 2011). Mice developed SCLC when they were intranasally (IN) injected with Ad-CMV-Cre, but selective targeting of club or AT2 cells did not promote SCLC under the conditions used. Instead of adenovirus, Park et al. (2011) used mice carrying Cre under the endogenous promoter of the CC10 marker to induce *Rb1* and *Trp53* deletions in club cells; to target AT2 cells, they used either adenovirus carrying CreER under the SPC promoter (Ad-SPC-CreER) or a SPC-rtTA/(tetO)7-Cre mouse line. These models did not yield any NE tumors from club or AT2 cells but only LUAD in a few cases. Differences between the reported experiments can be related to the different approaches used to induce genetic lesions, the length of time the animals were monitored, the influence of the genetic background, or environmental factors, such as local damage or inflammation that can influence lung cell transdifferentiation (Zheng et al. 2013). Nevertheless, Song et al. (2012) confirmed that SCLC can develop in lung NE cells in adult mice upon deletion of *Rb1* and *Trp53* using a CGRP-CreER knock-in allele (*CGRP^CreER/+^;Trp53^F/F^;Rb1^F/F^* mice). Similarly, loss of CREBBP (*Trp53^F/F^;Rb1^F/F^;Crebbp^F/F^* mice) (Jia et al. 2018) or overexpression of L-MYC (*Trp53^F/F^;Rb1^F/F^; invCAG-Mycl-Luc* mice) (Semenova et al. 2016) also give rise to SCLC upon infection with Ad5-CGRP-Cre as does deletion of *Rb1*, *Trp53*, and *Pten* (Cui et al. 2014; McFadden et al. 2014). Deletion of the latter gene set using Ad5-CMV-Cre results mostly in the development of acinar and mixed adenocarcinoma with neuroendocrine differentiation (Cui et al. 2014), suggesting that initiation from a different epithelial cell type alters the fate of cancer cells in this genetic context. Importantly, deletion of *Rb1* and *Trp53* and activation of *Myc* in NE cells using Ad5-CGRP-Cre were shown to result in the development of the *NeuroD1* variant form of SCLC (Mollaoglu et al. 2017), indicating that different subtypes of SCLC may arise from the same cell type. More recently, using *Ascl1^CreER/+^;Trp53^F/F^;Rb1^F/F^* mice and lineage tracing approaches, Ouadah et al. (2019) have suggested that SCLC originating from NE cells may actually arise from a subset of lung NE cells with stem cell features (NE^stem^). Whether NE^stem^ cells are the exclusive cell of origin for SCLC among all NE cells in the lung is currently unknown.

While NE cells or a subpopulation of NE cells are very likely to be a cell type of origin for SCLC, in line with the earlier data of Sutherland et al. (2011), recent findings have confirmed that SCLC can develop from various cell lineages, although with different efficiency. In a recent study, *Trp53^F/F^;Rb1^F/F^* mice injected with Ad5-SPC-Cre and, to a lesser extent, mice injected with Ad5-CC10-Cre were shown to develop SCLC. More strikingly, tracheobronchial basal cells, targeted with Ad5-K14-Cre, were identified as an additional potential cell of origin for SCLC (Ferone et al. 2020). In this model, the efficiency of transformation into SCLC as well as tumor latency matched that of mice injected with Ad5-CGRP-Cre, raising the possibility that a subpopulation of K14^+^ cells in lung is receptive to neuroendocrine transformation. Accordingly, deletion of *Rb1* and *Trp53* along with *Pten* and *Rbl1* (p107) with Ad5-K5-Cre, which targets cells similar to K14-expressing cells, also led to the development of SCLC, supporting the idea that basal cells can serve as a cell of origin for SCLC in the context of a different set of driver lesions (Lázaro et al. 2019).

Furthermore, certain mutations can modify the propensity of specific cell types to give rise to SCLC. This is the case for *Trp53^F/F^;Rb1^F/F^;Fgfr1^K656E^* mice, in which expression of a constitutively active form of FGFR1 in different lung compartments revealed a strikingly context-dependent effect. FGFR1^K656E^ selectively promotes SCLC from K14-expressing tracheobronchial basal cells but impairs SCLC development from CGRP-expressing NE cells (Ferone et al. 2020). Therefore, FGFR1 can act either as a driver or suppressor of SCLC, depending on the cell of origin.

In addition to modifying the tumorigenic effect of genetic drivers, the cell of origin also plays a role in tumor evolution and metastasis of SCLC as documented by Yang et al. (2018) in *Trp53^F/F^;Rb1^F/F^;Rbl2^F/F^* mice. Most of the metastasized SCLC found in these mice upon injection with Ad5-CMV-Cre exhibited high expression levels of the prometastatic transcription factor NFIB (Dooley et al. 2011; Semenova et al. 2016), whereas they were negative for NFIB when induced by injection with Ad5-CGRP-Cre. NFIB expression was shown to affect chromatin structure and augment accessibility by transcription factors (Denny et al. 2016). Hierarchical clustering of chromatin accessibility showed that primary SCLC and metastasis differed in this respect in Ad5-CMV-Cre-injected *Trp53^F/F^;Rb1^F/F^;Rbl2^F/F^* mice, whereas Ad5-CGRP-Cre-injected *Trp53^F/F^;Rb1^F/F^;Rbl2^F/F^* mice did not show any difference in chromatin accessibility between primary tumors and metastasis (Yang et al. 2018). How SCLC arising from CGRP-expressing cells disseminates and forms metastasis is still unknown and deserves further investigation. Regardless, these experiments clearly illustrate that tumor evolution is directed differently in tumors arising from CGRP-expressing cells and from one or more other cell lineages targeted by the ubiquitous CMV promoter. These different evolutionary pathways will likely also have an impact on how the tumors respond to therapy.

While the neuroendocrine phenotype and common occurrence of *RB1* loss in large cell neuroendocrine carcinoma of the lung (LCNEC) have suggested potential ties between this cancer type and SCLC (George et al. 2018), a robust mouse model of LCNEC development has not yet been developed. Recently, however, combining *Pten* deletion alongside *Trp53/Rb1/Rbl1* loss, which normally leads to SCLC development (Ng et al. 2020), has been shown to generate LCNEC in mouse models (Lázaro et al. 2019). Targeting the quadruple knockout to all cell lineages with Ad5-CMV-Cre resulted in a majority of tumors being LCNEC while targeting basal cells with Ad-K5-Cre resulted mostly in SCLC. Not only does this study represent the first and only mouse model for LCNEC reported so far, but it also further underscores that the cell of origin plays an important role in determining the lung cancer subtype specification even in the same genetic background.

A selection of relevant mouse models developing SCLC with specific emphasis on the targeted cell of origin, is summarized in Table 3 (see also Fig. 4). Information about the nature of the genetic lesions, the cell type specificity of the mutation inducer, and the tumor location are also included.

## Intratumoral heterogeneity

Intratumoral heterogeneity is a theme long believed to be specific for human tumors that arise through many steps driven by accidental lesions occurring at relatively high incidence as a result of DNA damage and chromosome instability. Interestingly, even in highly defined mouse models, where essential driver lesions are introduced by genetic engineering rather than inflicted by damage, there is substantial tumor heterogeneity (Ireland et al. 2020). The resulting intratumoral heterogeneity, which is likely to be driven by epigenetic changes in mouse models, is reminiscent of mechanisms that control normal tissue architecture with a guiding role for tissue stem cells. These cells depend on niches that provide the paracrine signals enabling their maintenance as well as their differentiation into a diversity of cell types with dedicated functions. This hierarchy is well defined for the hematopoietic system and for the intestine in which niche cells, such as Paneth cells, secrete Wnt to secure the maintenance of tissue stem cells (or in the context of a tumor, the tumor-initiating cells). Such paracrine interactions are also observed in other complex tissues such as lungs (Lee et al. 2017), and Wnt-producing niches are also critical for adenocarcinoma development (Tammela et al. 2017).

In the past, we provided evidence for the presence of clonal tumor populations in SCLC that are composed of both neuroendocrine and nonneuroendocrine cell types. Their paracrine interdependence was supported by the more effective proliferation and metastatic capacity of mixed cell populations upon subcutaneous grafting in mice (Calbo et al. 2011; Kwon et al. 2015). Whereas this specific study demonstrated that the paracrine effect of FGF2 produced by the nonneuroendocrine tumor cells on neuroendocrine SCLC resulted in the up-regulation of the ETS transcription factor PEA3, shown to be responsible for most of the effect of paracrine signaling, we also demonstrated that Notch signaling from neuroendocrine to nonneuroendocrine cells is an inherent feature seen in their SCLC model based on inactivation of *Rb1*, *Trp53*, and *Rbl2* (Lim et al. 2017). Notably, this intratumoral heterogeneity driven by Notch is less present in tumors initiated in CGRP-expressing cells compared with cells in which the CMV promoter is active, suggesting that the identity of the cell of origin can influence epigenetic heterogeneity, similarly to its effects on the mechanisms of metastasis discussed above.

While the interdependency of clonal subpopulations within lung cancers can contribute to intratumoral heterogeneity, tumors also promote heterogeneity through tumor-stromal interactions. Lung tumors recruit a diversity of stromal components, such as fibroblasts, immune cells, and vascular endothelial cells, as well as contribute directly to the tumor vasculature (Williamson et al. 2016). This vasculogenic mimicry has been observed in a number of other tumor types such as gliomas (Ricci-Vitiani et al. 2010). There is no reason to assume that this plasticity should be unidirectional. A tumor can consist of multiple different cell types, each with their specific vulnerabilities or refractoriness to distinct treatments but also capacity to interconvert, thereby creating a system in which a fraction of the tumor cells is likely resistant to treatment. This is still a relatively unexplored territory, and autochthonous mouse tumor models are the system of choice to better understand the rules and signals governing this plasticity (Tammela and Sage 2019). This cellular plasticity is likely also at the basis of the transdifferentiation, another source of heterogeneity. Tumor plasticity at an individual cell level may occur through genetic drift fostered by microenvironmental cues such as hypoxia or paracrine signaling from infiltrating immune cells or adjacent tissues. At a population level, the adaptive response of tumors may be the result of treatments in which the selective pressure, imposed by targeted drugs, selects for escape variants with very different phenotypic characteristics. The relapse of LUAD as SCLC is an illustrative example (Niederst et al. 2015). So far, such transdifferentiation has, to our knowledge, not yet been shown in mouse models. It certainly would be important to assess in more detail what the critical drivers of such transition are. Clearly, the loss of RB function is a critical requirement but seems insufficient by itself. Considering that AT2 cells serve as cell of origin for LUAD, the SCLC that results from inactivation of Rb and p53 in AT2 cells (see above and Table 3) may mimic some of steps required for such transition.

## Conclusion

The picture that transpires from studying the mouse models of lung cancer that have been developed is that of a highly versatile system in which multiple cell types in the lung can give rise to various lung cancer subtypes. Cell of origin and tumor subtype are clearly connected but with quite some infidelity. Specific drivers can facilitate particular subtype transitions or might block them. LSCC can be effectively generated from basal epithelial cells, AT2 cells, and club cells. This requires distinct driver lesions, and the transition from AT2 cells to LSCC follows a well-defined path in which the AT2 cells first starts to express club cell-specific marker CC10 with concomitant down-regulation of the AT2-specific marker SPC. Subsequently, the cells lose CC10 expression and become P63^+^ and K5^+^, which are the characteristic markers of LSCC. The tumors originating from these different cells of origin are indistinguishable based on RNA expression profiles, although it is not excluded that they retain specific epigenetic imprints from the cell of origin.

LUAD is most efficiently induced from AT2 cells and, to a lesser extent, from CC10^+^ cells localized at the BADJ region. Tumors arising from this latter location have a clearly different, more papillary phenotype. Here, proliferative lesions positive for SOX2 and CC10 and negative for SPC lose these markers and become SPC-positive. Basal epithelial cells do not appear to serve as effective cells of origin of LUAD. The phenotype of LUAD is also strongly influenced by transcription factors such as NKX2-1 and FOXA1/2, or LKB1, the loss of which can push LUAD to adenosquamous cell phenotypes. Similarly, treatment of mutant-EGFR LUAD with EGFR inhibitors can result in the transdifferentiation of LUAD into SCLC with concomitant changes in drug sensitivities.

SCLC can be induced from a variety of cells, with NE cells being an effective cell of origin upon loss of *Rb1* and *Trp53*. However, targeting *Rb1* and *Trp53* loss to basal epithelial cells also appears as an efficient route to SCLC. Interestingly, activation of FGFR signaling—regularly seen in human SCLC—is well tolerated when SCLC is induced from basal epithelial cells, whereas FGFR signaling potently inhibits SCLC initiated from NE cells. There are also peripheral cells that do not express K14, CC10, SPC, or CGRP, but nevertheless give rise to SCLC-like neuroendocrine tumors. These tumors are particularly prominent when *Rb1* and *Trp53* loss induced by Ad5-CMV-Cre is combined with overexpression of *Mycl.* The tumors resemble the lesions induced by Ad5-CMV-Cre in *Trp53^F/F^;Rb1^F/F^;Rbl2^F/F^* mice (Yang et al. 2018). These neuroendocrine tumors, with close resemblance to SCLC, exhibit a clearly different expression pattern and show intrinsic resistance to chemotherapy regimens, thereby serving as an illustrative example of how the cell of origin can modulate critical tumor features relevant for therapy.

Overall, mouse models of lung cancer can teach us important lessons about the cells of origin of lung tumors and the driver lesions and/or epigenetic modulations needed to permit a particular cell to act as the cell of origin. Given the close resemblance with the cognate human lung tumor subtypes, many of the lessons learned might also be applicable to human lung tumors and it will be important in the future to model subtypes of lung cancers that have not been modeled yet in mice. Furthermore, mouse models can also provide insight into the role of stage-specific drivers that might be required only at a particular phase of tumor development (e.g., being irrelevant during tumor progression and, consequently, being unsuitable as target for intervention). A detailed inventory of the expression profiles of all cell types of lung from both humans and mice (Tabula Muris Consortium 2018) will allow us not only to correlate normal cell types between both species but also to identify specific lineage markers that can help to trace back the cell of origin of tumors. Applying organoid and 3D whole organ imaging with immunolabeling at single cell resolution (Rios et al. 2019) can greatly help in understanding tumor architecture and biomarker expression. This will facilitate comparisons with human lung tumor samples analyzed by single cell sequencing techniques (Laughney et al. 2020) to understand the underlying biology that will remain of crucial importance for developing more effective therapies.
